# The emerging links between chromosomal instability (CIN), metastasis, inflammation and tumour immunity

**DOI:** 10.1186/s13039-019-0429-1

**Published:** 2019-05-14

**Authors:** Andréa E. Tijhuis, Sarah C. Johnson, Sarah E. McClelland

**Affiliations:** Barts Cancer Institute, Queen Mary University of London, EC1M 6BQ, London, UK

## Abstract

Many cancers possess an incorrect number of chromosomes, a state described as aneuploidy. Aneuploidy is often caused by Chromosomal Instability (CIN), a process of continuous chromosome mis-segregation. CIN is believed to endow tumours with enhanced evolutionary capabilities due to increased intratumour heterogeneity, and facilitating adaptive resistance to therapies. Recently, however, additional consequences and associations with CIN have been revealed, prompting the need to understand this universal hallmark of cancer in a multifaceted context. This review is focused on the investigation of possible links between CIN, metastasis and the host immune system in cancer development and treatment. We specifically focus on these links since most cancer deaths are due to the consequences of metastasis, and immunotherapy is a rapidly expanding novel avenue of cancer therapy.

## Introduction

For years, the main focus of cancer research has been on identifying genes that seem to have an influence on tumourigenesis (oncogenes, tumour suppressor genes, and DNA repair genes), and subsequently developing targeted therapies against the products of these genes. This has led to the development of some successful cancer therapies. However, it has also become apparent that the development and progression of cancer does not exclusively rely on the mutation of single genes. It has long been known that a large proportion of cancers are aneuploid, and many cancers also display Chromosomal Instability (CIN). Historically, specific aneuploidies have long been known to be linked to an improved or worsened prognosis, in many cancers including leukaemias [1–3]. The existence of aneuploidy and/or CIN in tumours often leads to large-scale genetic changes, changing the expression of many genes at once. Targeting one or a few gene products in cancer might be too simplistic an approach, given the often-complex genotypes of tumours. For this reason, aneuploidy and CIN have received more interest in the last few years. In particular, intriguing connections between CIN, metastasis, inflammation and tumour immunity are emerging themes. Here we discuss research in these areas to provide a global overview of the importance of CIN including, and beyond, its canonical role in promoting tumour genomic diversity.

### Aneuploidy and chromosomal instability

Aneuploidy is defined as the state of having an amount of chromosomes that deviates from a multiple of the haploid number [4]. Healthy human cells contain two sets of 23 chromosomes, one set inherited from each parent, totalling 46 chromosomes (a state also called ‘euploid’). Human cells with 47 or 45 chromosomes would be considered aneuploid. If cells have gained a full extra set of chromosomes (and therefore have 92 chromosomes), they are not considered aneuploid, but polyploid [4]. One well-known instance of aneuploidy among humans is Down syndrome, where affected individuals have three copies (a trisomy) of chromosome 21 instead of two (disomy).

**Fig. 1:**
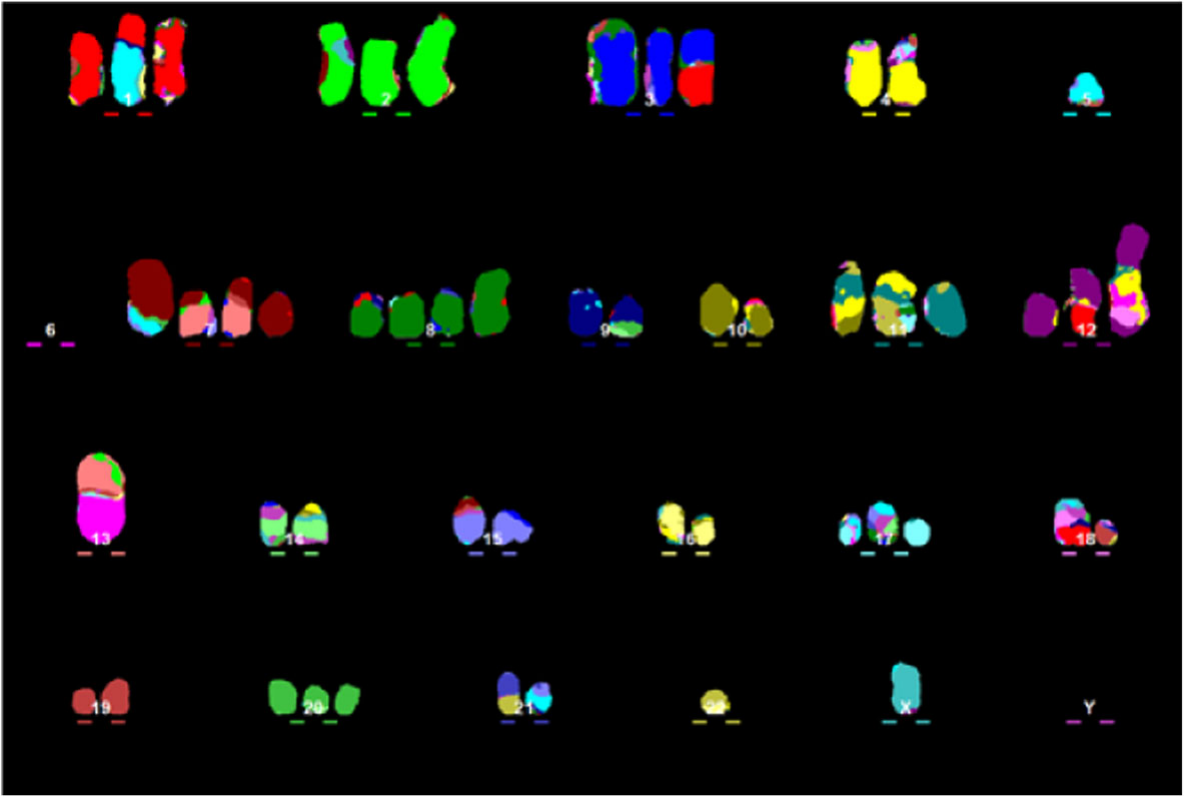
A karyogram from a high grade serous ovarian carcinoma cell (Kuramochi) showing extensive numerical (e.g. chromosome 3, green) and structural (e.g. chromosome 1 (red) translocation to chromosome 5 (turquoise) aneuploidy

Aneuploidy itself can be notionally subdivided into numerical or structural chromosome aberrations, often termed numerical or structural aneuploidy. Numerical aneuploidy is defined as having gains or losses of whole chromosomes, therefore inducing a change in the *number* of chromosomes [4]. When a cell suffers gains, losses, or translocations of parts of one or multiple chromosomes, it is called structural aneuploidy, as it changes the *structure* of chromosomes without necessarily changing the number of chromosomes [4] (Fig. 1). These states can exist separately from each other, for example in Down Syndrome (numerical only), or leukaemias with a single chromosome translocation (structural only), but they are not mutually exclusive [5]. In fact, in the majority of cancer types numerical and structural aneuploidy are exquisitely intertwined (see Fig. 1), suggesting possible mechanisms that can simultaneously promote both types of aberration [6, 7]. One interesting exception to this rule is neuroblastoma, where numerical aneuploidy can occur alone, and this class of aneuploidy confers a significantly better prognosis when compared to structural aneuploidy, or a combination of structural and numerical aneuploidy [8].

While aneuploidy is a *state* of abnormal chromosome number, chromosomal instability (CIN) is the heightened *rate* of the acquisition of chromosome abnormalities [9]. It is possible for cells to display aneuploidy without CIN, exemplified by individuals with Down syndrome. Interestingly, aneuploidy can, in some cases, also induce CIN. Experiments found that cells with a single chromosome addition often displayed more subsequent chromosome gains or losses [10]. Moreover, these cells displayed causes and/or characteristics of CIN, such as (ultrafine) anaphase bridges [10], micronuclei [11], chromosome mis-segregation and cytokinesis failure [12]. Aneuploidy in itself therefore also seems to be a possible ‘gateway’ to increasingly elevated CIN. However, CIN is generally held to lead to aneuploidy, as the effects of CIN will invariably lead to structural and/or numerical aneuploidy.

**Fig. 2:**
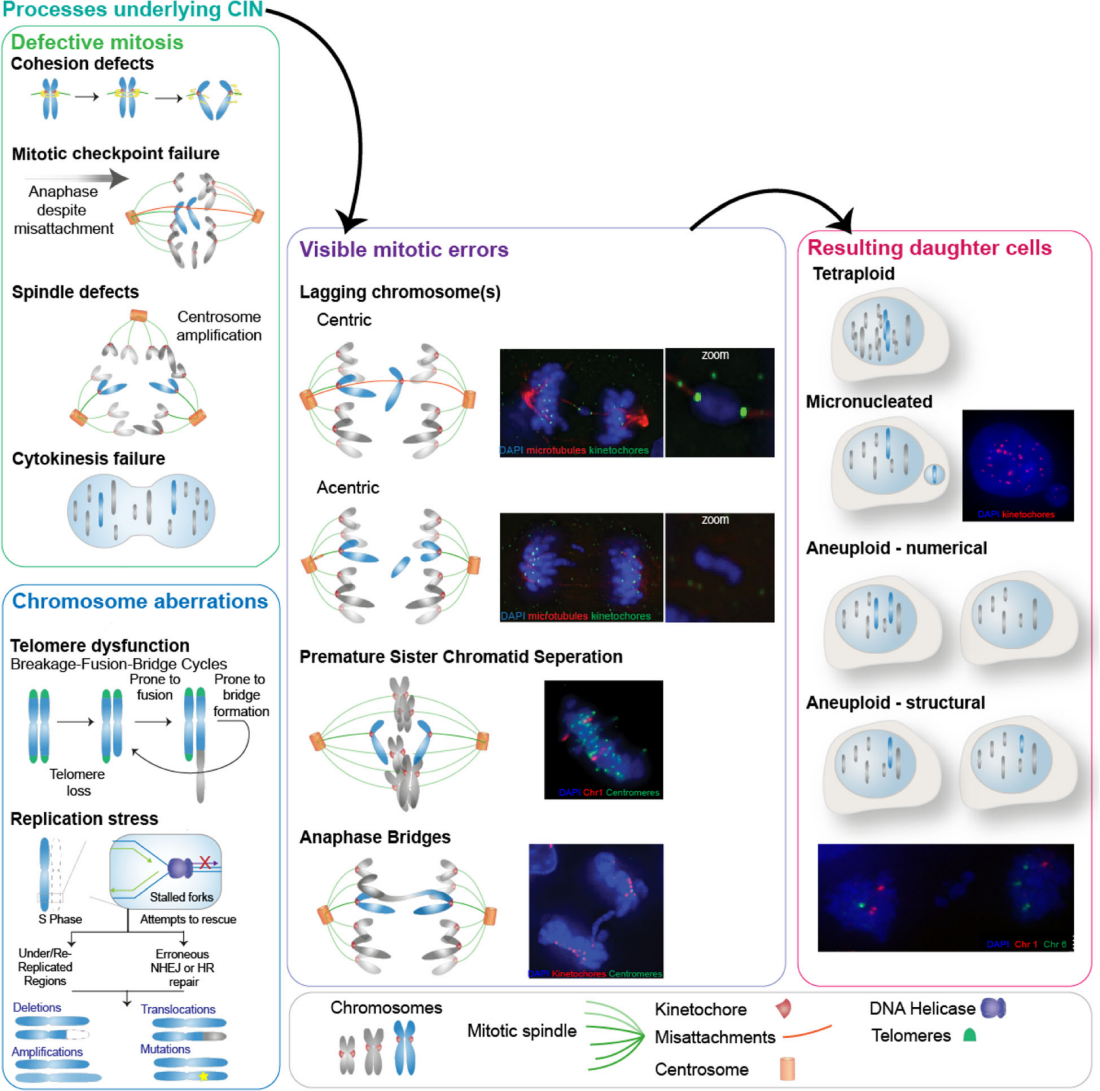
Mitotic and chromosome replicative defects that can lead to CIN. Common defects in mitosis leading to CIN include cohesion defects, spindle assembly checkpoint dysfunction, supernumerary centrosomes and cytokinesis failure. Problems upstream of mitosis, during DNA replication and repair can include telomere dysfunction, leading to breakage-fusion-bridge (BFB) cycles, and replication stress

The generation of both structural and numerical aneuploidies can be visible as mis-segregated chromosomal material during mitosis, where duplicated chromosomes are divided between two new daughter cells. Mitosis is a tightly regulated process with number of key proteins involved in assuring accurate segregation of chromosomes to their daughter cells. However, errors in mitosis can still occur (e.g. due to mutation of a mitotic regulator), and some of these mitotic errors can lead to aneuploidy [4, 9]. For example, mistakes in attachment of spindle microtubules to a chromosome leads to mis-segregation of that chromosome, resulting in a numerical aneuploidy. Alternatively, attachment of multiple spindle microtubules from opposing sides/daughter cells can effectively ‘tear apart’ a chromosome, causing the two daughter cells to either gain or lose part of that chromosome, leading to structural aneuploidy (Fig. 2). While aneuploidy becomes apparent during mitosis, it can be caused by processes preceding mitosis, for example DNA damage or replication stress [6, 7, 13]. There are multiple defects that have been proposed to underlie CIN in cancer: e.g. mitotic checkpoint errors [14], lagging chromosomes [15], anaphase bridges [16, 17], mono- [18–20] and multipolar [21, 22] spindles, cytokinesis failure [23], telomere dysfunction [24, 25], replication stress, and DNA damage (**see** Fig. 2), which has been covered extensively elsewhere [6, 14–25]. An important point to note however, is that determining mechanisms of CIN from patient tumours is notoriously difficult. Furthermore, many studies focus on cell lines from a diverse panel of tumours, whereas CIN mechanisms are likely to differ between tumour types, and potentially patients (our unpublished observations). In addition, recent research found that chromosome mis-segregation has a non-random pattern in non-transformed cells, with some chromosomes mis-segregating significantly more often than others [26]. Common Fragile Sites (CFSs) are sites within chromosomes that are known to be more prone to breakage when cells experience replication stress, leading to structural aneuploidy [27]. These observations show that both numerical and structural aneuploidy could be generated at non-random genomic sites; the possibility thus exists that this pattern is different in different types of cancers as a result of distinct chromosomal instability mechanisms.

In addition to chromosomal changes in the nucleus of the cell, CIN can also promote the acquisition of abnormal cellular structures, such as micronuclei. Micronuclei are small nuclear structures containing a small amount of genetic material. This genetic material can be a single chromosome that was lagging during chromosome separation or a fragment of broken DNA resulting from DNA damage [28] (Fig. 2). Micronuclei contain less DNA repair and replication machinery, and are often prone to rupture, increasing the further accumulation of chromosomal abnormalities [28, 29]. They can also activate the innate immune system due to release of DNA into the cytoplasm (see below).

### The consequences of aneuploidy and CIN in healthy cells and cancer

Aneuploidy usually brings about drastic changes in the genetic component of a cell. As can be expected, aneuploidy normally not well-tolerated in individuals. This is especially shown by the spectrum of congenital aneuploidies in humans: almost all congenital aneuploidies are lethal in utero (the most notable exception being trisomy 21) [30], and many spontaneous abortions contain aneuploidies [31]. Structural congenital aneuploidies can have detrimental effects on health, depending on the location and size of the structural abnormality [32, 33]. Unsurprisingly, characteristics of CIN are also poorly tolerated. The Spindle Assembly Checkpoint (SAC) is an important mechanism ensuring correct chromosome segregation [14]. Mutation of the gene encoding an important component of the SAC, BubR1, can lead to Mosaic Variegated Aneuploidy (MVA), a condition characterised by mosaic aneuploidy caused by increased chromosome mis-segregation [34, 35]. Experiments conducted on mouse embryos revealed that BubR1 deficiency can lead to embryonic lethality [36]. In humans, individuals born with this congenital condition grow at a slower rate compared to healthy individuals, and display a range of other abnormalities. In addition, individuals with MVA also have a higher risk of developing childhood cancers [34, 37]. Consistent with the detrimental effects of aneuploidy on whole organisms, it has been shown that aneuploidy generally causes reduced fitness in cell populations [30]. The antiproliferative effects of aneuploidy have not only been shown in yeast, but also in mammalian cell culture and in vivo [38–40]. The reduced fitness of aneuploid cells imposes selection pressure favouring euploid cells. Research by Pfau and colleagues used hematopoietic stem cells (HSC) with increased rates of chromosome mis-segregation and subjected them to serial transplantation experiments [40]. Karyotyping of the reconstituted HSC population showed that no aneuploid cells were present, despite the increased rate of chromosome mis-segregation present in these cells, indicating that aneuploid cells are selected against in vivo [40].

Paradoxically, despite the observation that aneuploidy is an unfavourable state for healthy cells, this does not seem to be true for cancer cells. In contrast to normal cells, aneuploidy is a characteristic displayed by the majority of cancers: as many as 90% of solid cancers and 50% of haematopoietic cancers are aneuploid [30]. Because aneuploidy seems to be ubiquitous in cancer, a large amount of effort has been dedicated to examining the link between aneuploidy and tumourigenesis. However, the precise relationship between the two is still not completely understood [41]. As aneuploidy is normally detrimental to cell health, it was suggested that aneuploidy is not a driver of tumourigenesis, but rather a passenger event. Research over the years has yielded inconclusive results, both supporting and contradicting the hypothesis that aneuploidy can lead to cancer. Investigations on cancers and their premalignant counterparts have shown that aneuploidy is already present in the precursors, but increases with progression to malignancy [42]. This has also been shown with CIN [43, 44].

As with aneuploidy, many tumours also display a higher rate of CIN. A popular hypothesis for the high incidence of CIN and aneuploidy in cancer cells is that CIN continually generates ‘new’ karyotypes, increasing intratumour heterogeneity (ITH). This increases the possibility of the emergence of, and selection in favour of, an advantageous karyotype. For example, monosomy of chromosome 3 is often seen in uveal melanoma and describes a more aggressive subtype [45]. Several experiments have also shown that inducing CIN (for example by deregulating elements of the mitotic checkpoint) can lead to tumourigenesis [46, 47]. Interestingly, while intermediate levels of CIN seem to induce tumourigenesis, high CIN has been shown to impair tumour growth and improve patient prognosis [48–50]. In instances of very high CIN, the generation of unviable karyotypes might outweigh that of possibly advantageous ones, leading to impaired tumour proliferation [51]. Importantly, p53 deficiency can increase the tumourigenic potential of CIN [39]. This is likely due to the negative consequences of CIN, such as aneuploidy, that are detrimental to cell viability and often lead to p53 activation [20, 39, 52]. In order to sustain CIN, p53 expression is therefore lowered or abrogated in tumours exhibiting CIN.

### The relationship between CIN/aneuploidy and genetic intratumour heterogeneity (ITH)/Cancer evolution

Intratumour heterogeneity (ITH) is often described as the presence of a large variety of differently behaving cells within a tumour [53]. Many tumours display ITH, which can be genetic, but can also indicate phenotypic differences within a tumour due to spatial distribution, among other factors [54–58]. Different genetic makeup between subclones may be the result of chromosomal instability, and genetic/chromosomal instability has long been a proposed mechanism of ITH [53]. An important question is why ITH is so often seen in tumours, and if and how it could impose a survival advantage over a homogeneous population. The most popular theory is that ITH, induced by genomic instability, accelerates cancer evolution [59, 60]. The role of CIN in the metastatic progression of cancer might be due to the enhanced adaptive properties it imposes on tumour cells, which will be further explored below.

#### Aneuploidy confers selective advantage under stress conditions

During cancer development, the tumour population is put under severe intra- and extra-cellular stress. Because of these evolutionary stresses, the population continuously needs to change in order to select the fittest clones. This change could also come in the form of a novel karyotype. Indeed, some aneuploid karyotypes seem to be better suited to particular stress situations. In vitro experiments in yeast used euploid and a number of aneuploid yeast cell populations in which a stable, but aneuploid karyotype was experimentally induced. These cells were exposed to unfavourable conditions, and it was seen that some of the aneuploid populations had a higher fitness than the euploid cell population [61]. The ‘fittest’ karyotype varied based on the specific cellular stress. Importantly, this fitness advantage was not seen under normal growth conditions [61]. Such an aneuploid fitness advantage has also been observed in subsequent yeast experiments [62–64]. In vitro research performed by Rutledge and colleagues found compelling evidence for the selective advantage of aneuploid karyotypes in human cells [65]. The researchers cultured both diploid and trisomic CRC cells (derived from the same cell line, trisomic for either chromosome 7 or 13). It was found that the aneuploid cells had a growth advantage over diploid cells under some stress conditions, but not standard conditions [65]. Interestingly, aneuploid cell populations were found to exhibit enhanced invasive properties, irrespective of their specific trisomy, even under standard growth conditions [65].

#### CIN is an important player in cancer evolution

While aneuploid karyotypes can be generated in the absence of active CIN driver mechanisms (e.g. a single, stochastic mis-segregation event), CIN is a much more efficient way of generating possibly advantageous aneuploid karyotypes. A reason why CIN is so prominent in cancer is that it is a major factor in cancer evolution. As has become clear from the previous chapter, CIN is able to induce a range of genomic changes, ranging from subtle to massive. Examples of large genomic changes that can be ascribed to CIN are chromothripsis and genome chaos [66–68]. Such large changes are often needed during cancer development, as the tumour experiences stresses that need to be overcome in order to survive; such stresses include chemotherapy and immune pressure) [69, 70]. In such a context, rapid genome-wide changes are often more effective in inducing adaptation than single gene mutation, as large genomic rearrangements/changes lead to a change in genome organisation, interactions between genes, and the transcriptome, therefore leading to a significant change in phenotype [71]. While not every genomic rearrangement leads to a viable karyotype, the added ITH introduced by CIN is thought to increase the probability of a viable karyotype within the population [70]. During cancer evolution, tumour cell populations with a high degree of CIN and a large variety of distinct karyotypes could be able to drive rapid stress adaptation. Such examples of events of punctuated evolution or macroevolution have been observed in cancer [72–74]. Following a burst of punctuated evolution, subsequent single gene mutations could then further fine-tune the selected karyotypes to reach optimal growth and survival (for a more in-depth review on the relationship between CIN and cancer evolution, see References [71, 75]).

In yeast, cellular stresses themselves have also been seen to induce CIN which, in turn, leads to stress adaptation by karyotype diversity [76]. In addition, research based on ER-negative breast cancer found evidence for the selection of amplifications of proliferative genes through CIN [77]. A recent in silico simulation of CIN cell populations performed by Laughney and colleagues provides evidence that CIN could indeed play a large role in cancer evolution [78]. It was found that cell populations in these simulations worked towards an ‘optimal’ near-triploid karyotype. Strikingly, database analysis found that this ‘optimal’ number of chromosomes is also regularly seen in patient tumour samples [78]. Sotillo and colleagues performed in vivo research in a background of inducible oncogene (KRAS) amplification with or without CIN (induced by Mad2 overexpression) [79]. Upon oncogene withdrawal, the tumours in these mice regressed. Interestingly, recurrence of tumours was seen in mice exhibiting CIN, but not those without CIN. It was proposed that CIN lead to the development of subclones that did not depend on expression of the oncogene, thereby escaping oncogene withdrawal. This has also been shown in a follow-up study by Rowald and colleagues [80]. Taken together with the results discussed in the previous section, these lines of evidence form an argument that CIN can indeed fuel cancer evolution, by providing a means for tumour cells to adapt under selection pressure, thereby being able to persist in unfavourable conditions.

### CIN and metastasis

Metastasis has received an abundant amount of attention in cancer research, and this is with good reason. Metastasising disease is the number one cause of cancer mortality, being responsible for approximately 90% of cancer-related deaths [81]. It is therefore of particular importance to uncover the mechanisms by which cancer is able to metastasise, in order to be able to develop possible therapy. (see Table 1).

**Table 1:** Metastasis

Metastasis is the spread of a cancer to tissues other than the site of the primary tumour where the cancer originated. The ability to metastasize has been defined as a hallmark of cancer in Hanahan and Weinberg’s original review [82]. Metastasis is often the cause of cancer-related deaths. As such, metastasis is used as a criterion to classify tumour stage. Metastases are often already present when a patient is first diagnosed. In order to metastasise, tumour cells need to go through a stepwise process, which involves being able to ‘break free’ from the primary site, enter into the lymph and/or blood circulation, exit the circulation, and settle and proliferate at the site of metastasis. Metastasising cancer cells need to satisfy the conditions for each step before successfully invading another tissue; this often requires a genetic adaptation. Several genetic adaptations have been associated with one or more of the steps of metastasis. For example, loss of the adhesion protein E-cadherin has been found to increase tumour invasiveness, as cells lacking E-cadherin expression are more loosely connected to other cells, increasing their motility [83, 84]. Another pathway associated with metastasis is the Epithelial to Mesenchymal Transition (EMT), where an epithelial cell gains the characteristics of a mesenchymal cell, such as loss of cell polarity and enhanced motility and invasion [85, 86]. Metastatic cancer cells have also been observed to possess invadopodia, cellular structures that aid in extravasation [87]. One of the main discussion points around metastasis is how it originates and at what point in tumourigenesis it arises [88]. Metastatic cells were first thought to arise from the primary tumour in a linear fashion: a subset of cells from the primary tumour accumulate metastasis-promoting mutations over time, until a subclone with full metastatic potential arises [89]. This metastatic subclone would thus arise late in tumourigenesis and therefore have substantial genetic similarity to the primary tumour. More recently, a different model for the generation of metastases was proposed. This parallel progression model argues that a metastatic subclone is formed and disseminates early during tumourigenesis [90]. This clone might not be fully metastatic (i.e., not yet capable of distant organ colonisation) but acquires these characteristics separately from the primary tumour. Thus, in this model the similarity between primary tumour and metastasis is expected to be lower. Research on primary tumours and their metastases has generated results supporting both theories [91–95], and modes of metastases might differ between cancer types or even between patients [96, 97]. There is as of yet no consensus on whether the mode of progression depends on the cancer type and environment. Another point that has recently come under debate is how cancers exactly spread to distant organs. Lymph node metastases are often seen before distant metastases, and this has led to the assumption that distant metastases are derived from the lymph node metastases. This paradigm is questioned by a recently published paper by Naxerona and colleagues. From their phylogenetic investigation of lymph node and distant tumours, it follows that only 35% of distant metastases are derived from lymph node metastases [98]. The cellular characteristics needed for lymph node involvement might therefore be different from those needed for distant spread. Still, lymph node involvement is a robust prognostic factor for the development of distant metastases in many cancers, so LNM does tell something about the metastatic potential of a primary tumour.	

o

#### CIN as a prognostic factor in metastatic cancer

One of the first questions one may ask about a possible link between CIN/aneuploidy and metastasis is whether aneuploid tumours are more often metastatic, or whether metastases are more often aneuploid. As metastasis is known to be responsible for most cancer deaths, an indirect way of approaching this question would be to assess whether patients with aneuploid primary tumours have a worsened prognosis for overall survival. Indications for a link between primary tumour aneuploidy and prognosis have been in existence for several decades, where patient studies found that aneuploid tumours were often of a higher grade and stage and were associated with decreased patient survival in several types of cancer [99–105]. This link has also been found in more recent investigations [106–115]. A meta-analysis of 141,163 breast cancer cases performed by Xu and colleagues showed that patients with aneuploid breast cancer had worsened survival compared to patients bearing a diploid tumour [116]. In addition, aneuploidy was also associated with breast cancers containing lymph node metastasis [116]. Worsened prognosis does not seem to be associated with a specific type of aneuploidy, although particular numerical [45] and structural [117, 118] aneuploidies have been linked to a worsened prognosis in specific cancers. However, other studies failed to uncover a link between aneuploidy and worsened prognosis [119, 120]. Authors of these papers suggest that such discrepancies might have arisen from differences in population selection (e.g., only young patients monitored after curative resection). Still, these results also need to be considered when formulating a hypothesis about the prognostic effect of aneuploidy.

#### Aneuploidy and the occurrence of metastasis

In addition to assessing aneuploidy’s prognostic value, some studies have also looked into whether aneuploid tumours were found to metastasise more often. A meta-analysis on colorectal cancer (CRC) performed by Laubert and colleagues indicated that aneuploidy was linked to higher tumour stage in the majority of the studies analysed [121]. A recent study on endometrial cancers did not find any link between primary tumour ploidy and lymph node metastases (LNM) [122], while other studies on endometrial cancer did indicate that aneuploidy was predictive for LNM [118, 123]. Some investigations on gastric cancer found an increase in aneuploidy in distant metastases [124]. In summary, there seems to be slightly more evidence in favour of a relationship between aneuploidy and the occurrence of metastasis, but this is by no means clear cut overall.

#### Clinical research on CIN, prognosis, and metastasis

Despite its known association to cancer, CIN has received less interest in clinical association studies when compared to aneuploidy. One of the reasons for this is that it is more complicated to assess and quantify CIN in tumour cells. CIN is a dynamic process leading to a higher rate of chromosome mis-segregation and varying levels of aneuploidy between cells. To assess CIN, one must compare cells within a cell population or look at a cell population for an extended period of time [125]. Therefore, in association studies, CIN is sometimes, somewhat inaccurately, defined as the presence of aneuploidy as determined by flow cytometry [126]. Most research does find an association between CIN and prognosis, albeit using a range of different methods to assess CIN [126–129]. Some research also indicated an increased incidence or rate of CIN in metastases compared to primary tumours [127, 130]. One of the most comprehensive analyses on the association between CIN and prognosis is the analysis performed by Carter and colleagues [131]. They determined a gene set whose expression correlated with occurrence of aneuploidy, and from this derived a ‘CIN signature’. It was found that tumours with a higher correspondence to this CIN signature had a worse prognosis than those with a lower CIN signature [131]. Most interestingly, metastases were also found to have a higher CIN signature than primary tumours. It has been known that low or intermediate levels of CIN seem to bestow cancer cells with a growth advantage, but extreme CIN seems to have the opposite effect [48, 49]. Research using the same CIN signature showed that cancers with the worst prognosis were not the ones with the highest CIN signature, but the ones with an intermediate-high CIN signature [50, 132].

#### Ploidy differences between primary tumours and their metastasis

Assessing ploidy differences between primary tumours and their metastases might be important, as this could show whether aneuploidy only promotes the development of metastasis, or that aneuploidy is necessary or beneficial for survival outside the primary tumour stroma. Alternatively, it could show that change in stroma influences ploidy. Such ploidy assessment in pairs of primary tumours and their metastases has been performed less extensively. Several analyses of CRC have shown that the degree of aneuploidy in metastases is similar or heightened in comparison to the primary tumour [133–136]. In contrast, some investigations show that metastases seem to be diploid more often than the primary tumours they originated from [137–140]. A recent investigation comparing primary oral squamous cell carcinomas (OSCCs) and their lymph node metastases (LMNs) found similar rates of aneuploidy in primary tumours and lymph node metastases [141]. Strikingly, it was found that an equal amount of LNMs displayed either increased or decreased aneuploidy when compared to their primary counterpart [141]. Another study on OSCC found that while most LNMs displayed similar levels of aneuploidy in comparison to the primary tumour, 13% of LNMs showed a decrease in ploidy (going towards diploidy) and 3% of LNMs showed increased ploidy [142]. Finally, recent research conducted by Bloomfield and Duesberg specifically looked into the relationship between aneuploidy and metastasis. To this end, they compared the karyotypes of cell lines derived from seven aneuploid primary tumours and their metastases [143]. Their results indicate that all metastases show karyotypes similar to their primary counterpart, but distinct from metastases of other tumours. In addition, because all of the primary-metastatic tumour pairs display a karyotypic change, the researchers argue that the potential to metastasise is generated by a karyotypic change, rather than an enabling mutation [143]. However, this research does not directly show that aneuploidy itself is responsible for metastasis, as the researchers argue. All primary tumours investigated already displayed aneuploidy to some degree, and no comparison was made between aneuploid and diploid primary tumours and their respective metastases. It is therefore difficult to assess the degree of involvement aneuploidy has in metastatic spread in this case. In addition, the researchers’ statement that mutations themselves do not influence metastasis might be too extreme, as mutations are known to play a big part in tumourigenesis and cancer progression.

#### Indirect associations between CIN and metastasis

It is possible that factors linked to CIN might also indirectly promote metastasis. For example, Godinho and colleagues found that centrosome amplification provided cells with an invasive phenotype in vitro [144]. These cells were found to have invasive cell protrusions and looser cell-cell adhesion, which are properties needed for cells to metastasise. Centrosome amplification is associated with CIN [145] but in this case, CIN did not directly drive invasion. Centrosome amplification has also been clinically associated with poor prognosis and metastasis [146, 147]. Secondly, research conducted on Drosophila developing wing discs found that inducing CIN through inhibition of the SAC protein Bub3 caused epithelial cells to become highly motile [148]. These high CIN cells were seen to delaminate from the basal layer and invade the neighbouring wing disc compartment. This behaviour seemed to be mediated by activation of components of the EGFR and JNK pathways. In another example, mutation of DAXX and ATRX was found to induce CIN and lead to a worsened prognosis and metastasis [149]. Additionally, research on cancers of unknown primary origin (CUP) found that CUP had a higher rate of CIN than metastasis with known origin [150]. This is interesting, as CUP are considered to be metastases themselves. Recent research showed that mutation of the DNA repair gene ATM induced chromosomal instability in a pre-existing mouse model of pancreatic cancer [151]. ATM has been previously linked to suppression of aneuploidy [152]. Interestingly, ATM mutation also caused a higher frequency of metastasis. Moreover, more non-clonal chromosome alterations were found in these metastases compared to the ATM-proficient metastases [151]. The authors hypothesised that chromosomal instability could be a driver of metastasis. In conclusion, in clinical studies CIN does seem to be associated with a worsened prognosis and metastases, although the level of CIN can cause differential effects and is thus important to consider.

In summary, there is a considerable amount of indirect evidence that points towards an association between aneuploidy and the formation of metastasis. The majority of studies looking into prognostic factors for several types of cancer do find a correlation between aneuploidy and worsened prognosis. The relationship between aneuploidy and a higher probability of metastasis has been investigated less extensively. Although some results show evidence for the existence of this link, the research is still divided on whether aneuploid tumours metastasise more often than diploid tumours. Some research has looked into the possibility of ploidy differences between primary tumours and their metastases. Here, an even larger variance in results is found. Some studies show no difference in ploidy between primary tumour and metastasis, some show a higher incidence of aneuploidy in metastases, and yet others show a lower degree of aneuploidy in metastases. Finally, a thorough cytogenetic investigation of pairs of primary tumours and their (mostly distant) metastases shows that metastases display a very similar pattern of aneuploidy compared to the primary tumour. Collectively, the results of these papers do not provide any conclusive answer to whether or not metastases are more often aneuploid than their respective primary tumours. It may be that the contribution of aneuploidy and CIN varies with cancer type, or even within patients, thus more research is required to conclusively establish a direct link, or lack thereof, between CIN and metastasis.

### Potential mechanisms linking CIN to metastasis

Since there is likely an association between aneuploidy, CIN and the presence of metastases, what are the underlying mechanisms allowing these CIN tumours to metastasise? There are a few mechanisms known to promote metastasis in cancer. Firstly, cells capable of metastasis are thought to arise through a process known as cancer evolution. In this process, cells in a population continually change their genotype, and cells with an advantageous resulting phenotype are selected for (akin to normal evolution). One can understand that such cancer evolution happens more quickly in a population that is genetically heterogeneous and plastic. CIN is hypothesised to make a population more heterogeneous due to constant chromosomal changes. Metastases might therefore arise earlier in CIN tumours due to increased genetic heterogeneity.

#### CIN, ITH and metastasis

ITH is believed to lead to an overall growth advantage due to the enhanced adaptability of a heterogeneous tumour and has been determined to be present in different cancer types [55, 56, 58, 91, 153, 154]. While these effects of ITH have been the subject of numerous reviews, the adaptive benefit compared to a homogeneous phenotype has not been fully quantified. An experimental paper by Marusyk and colleagues shows indication for a growth advantage for polyclonal cell populations over monoclonal ones, and this largely seemed to depend on a single subclone [155]. Another study found that different subclones in a tumour can ‘support’ each other, causing the combination of subclones to be more aggressive than either subclone alone [156, 157]. In Barrett’s oesophagus, ITH is a prognostic factor for progression to malignant oesophageal adenocarcinoma [158]. Possibly the most compelling evidence that heterogeneity leads to adaptability is the emergence of resistant subclones after anticancer treatment. This has been linked to ITH in different cancer types, with some reports showing the presence of a resistant subclone in the primary tumour even before treatment [69, 159–161]. Presently, experimental papers linking intratumour heterogeneity and aneuploidy/CIN seem to be scarce. Research in yeast showed that aneuploidy increased phenotypic variation, even though the population was genetically stable [162]. Consequentially, the response to different stresses was also more variable than the response seen in euploid cells. This effect in variability was also shown in mouse embryos, which exhibited varying phenotypes for the same trisomy [162]. That aneuploidy seems to cause increased phenotypic heterogeneity in itself might indicate a key link between CIN and ITH.

As could be expected, heterogeneous tumours might also be able to select for clones exhibiting invasive behaviour [163]. Nevertheless, definitive experimental evidence for this association is limited. This might be due to the nature of ITH itself, as the presence of multiple subclones makes it more laborious to gather reliable clinical data from a limited number of biopsies per patient [91, 164]. Recently, advancements in multiregion sequencing have allowed more in-depth analysis of the heterogeneity within tumours [91]. This has also provided the potential to investigate the link between CIN and ITH, and their influence on metastatic progression. A recent study looked into ITH of Non Small-Cell Lung Cancer (NSCLC) [165]. The proportion of subclonal Copy Number Alterations (CNA) was correlated with disease recurrence and death, indicating that increased ITH has an effect on disease aggressiveness. The researchers used subclonal (mirrored) allelic imbalance assessment to determine the presence of CIN, and were able to detect such imbalances in 62% of tumours [165]. Such mirrored allelic imbalances reflect differences in chromosomal composition between subclones. This is therefore a measure for both ongoing CIN and ITH. The authors concluded that CIN did indeed seem to be linked to ITH and decreased survival. This was also shown in a very recent multiregion sequencing investigation of renal carcinoma by Turajlic and colleagues [166]. The researchers found evidence of selection in metastases, which were less heterogeneous than the primary tumours. A combination of both genomic instability and ITH served as a predictive tool for disease progression [166]. High genomic instability/CIN with low ITH showed rapid progression accompanied by a worsened prognosis, while intermediate genomic instability/CIN and high ITH supported a more gradual and less aggressive disease course. Interestingly, these results suggest that CIN does not always have to be accompanied by higher ITH. In contrast, a pan-cancer analysis by Andor and colleagues found that cancers with the best prognosis were those with low ITH and high levels of genomic instability (CNV burden) [154]. Another multiregion study performed on Triple-Negative Breast Cancer found that ITH could serve as a prognostic marker [167]. In this particular research, ITH was measured by calculating variance of the Copy Number Variations of several genes/gene areas (Myc, EGFR/CEP7, CCND1/CEP11) for each patient. It was found that high ITH as determined by EGFR/CEP7 or CCND1/CEP11 was significantly correlated with the development of metastasis [167].

It might currently be too soon to make any definitive conclusions about the influence CIN has on ITH and the subsequent promotion of metastasis. Aneuploidy, CIN, and ITH have been separately associated with metastasis. There are few studies investigating the combination of aneuploidy/CIN and ITH in relation to metastasis. Moreover, the conflicting results of papers published so far do not paint a clear picture of the exact relationship [154, 166, 167]. Recent research indicates that metastasis is a property selected for in cancers displaying both CIN and ITH [166]. However, from other research it seems that ITH is not necessarily an essential step or mechanism in cancer evolution, but more so a result of aneuploidy or CIN. Many papers demonstrate the adaptive properties of aneuploidy and CIN, and do not find heterogeneity, or do not take it into account. It is therefore still unclear if a certain level of ITH is needed to fully benefit from the adaptive properties of CIN, and utilise this for the selection of metastatic clones. Currently, ITH and CIN alone seem to be more strongly associated to metastasis than a particular combination of the two. As we are at the eve of the development of more sophisticated sequencing techniques, future studies might provide further evidence for the existence of a correlation between CIN, ITH, and metastasis.

### CIN and inflammation

Inflammation (in particular chronic inflammation) has long been known to influence tumourigenesis, and has been included as a (second-generation) hallmark of cancer [168]. It has been estimated that as many as 20% of cancers are caused by chronic inflammation [169]. Aside from being involved in tumourigenesis, inflammation and the innate immune system have also been associated with the promotion of metastasis [170–172]. The influence of aneuploidy on inflammation has been observed in cell lines with induced aneuploidy. These (mitotically arrested) cells were found to upregulate gene signatures associated with inflammation [51]. One of these signatures was the expression of cell surface proteins that are able to be recognised by Natural Killer (NK) cells [51]. While this might seem a way in which aneuploidy can promote metastasis, NK cells are known to have strong metastasis-suppressive effects and are being explored as possible anti-metastasis therapy [173–175]. Therefore, the recruitment of NK cells by aneuploid cells is unlikely to be the mechanism by which metastasis can be promoted. Indeed, the researchers showed that the aneuploid cells were effectively cleared by NK cells, while the euploid cells were not. The inflammatory response in aneuploid cells therefore mainly seems to serve as a mechanism for their clearing by the immune system. Of note, the inflammatory effects of aneuploidy have only been investigated in cell-cycle arrested cells in this research [51]. Possibly, aneuploid cells that do not experience cell-cycle arrest exhibit a different inflammatory response that helps them to persist. Aside from attracting NK cells, aneuploid cells were found to upregulate other inflammatory gene signatures. One of these upregulated inflammatory mechanisms that might be linked to CIN, and has recently received considerable interest, is the cGAS-STING (cyclic GMP-AMP synthase – Stimulator of Interferon Genes) pathway.

#### The cGAS-STING pathway linking CIN to inflammation

The cGAS-STING pathway has been described as a pathway responding to cytosolic DNA. cGAS has been identified as a DNA sensor, and has been shown to be activated in cells stimulated with cytosolic DNA [176]. These cells were then shown to produce IFN-β through the STING-mediated activation of IRF3 [176]. Cells are also seen to mount an innate immune response by DNA from DNA viruses, or that produced by reverse transcription of RNA from retroviruses. Importantly, this response is different to the one induced by viral RNA. Such a response was abolished upon knockdown of cGAS or STING [177]. cGAS-STING therefore seems to be an important component in the innate immune response against (retro)viruses [177]. The activation of the cGAS-STING pathway is currently believed to be as follows: DNA present in the cytosol (e.g. due to viral infection) is recognised by cyclic GMP-AMP synthase (cGAS), which produces cyclic GMP-AMP (cGAMP) that can activate Stimulator of Interferon genes (STING). As the name implies, STING is able to stimulate expression of interferons (IFNs) and, importantly, NF-κB (*see* Table 2), mainly by inducing the phosphorylation and subsequent activation of interferon regulatory factor 3 (IRF3, [189]).

**Table 2:** cGAS-STING and NF-κB

NF-κB (nuclear factor kappa-light-chain-enhancer of activated B cells) is activated by the cGAS-STING pathway and is an important player in inflammation-induced cancer. NF-κB comprises a group of transcription factors that regulate transcription of a large number of genes involved in many different pathways (e.g. growth and repair) by binding to the κB enhancer. NF-κB can be activated via two main routes, with each of the routes inducing different subunits and establishing different cellular effects [178, 179]. The first activation pathway is the canonical (or classical) pathway, and involves the NF-κB subunits p50 and p65. The second activation pathway is called the non-canonical (or alternative) pathway and involves the NF-κB subunit p52 (derived from p100 processing), as well as the RELB subunit. While canonical NF-κB activation is strong and transient, non-canonical NF-κB activation is slower and more often constitutive [179]. Canonical NF-κB activity includes the secretion of many inflammatory compounds, such as TNF and interleukins [178]. Non-canonical NF-κB activity is associated with lymphoid organ development, autoimmune T-cell removal, bone metabolism and B-cell maturation [179]. The important role of NF-κB in cancer promotion has been shown in two pioneering papers looking into Colitis-Associated Cancer and Hepatocellular carcinoma, respectively [180, 181]. NF-κB has an impact on many different pathways, and has been found to promote proliferation, angiogenesis, EMT, matrix degradation, and sustained inflammation [178, 182–186]. Moreover, inhibition of NF-κB induces antitumour effects and has been found to inhibit metastasis [187, 188]. As NF-κB is activated by cGAS-STING (among other pathways), it seems plausible that cGAS-STING-mediated NF-κB activation can promote metastasis.	

**Fig. 3:**
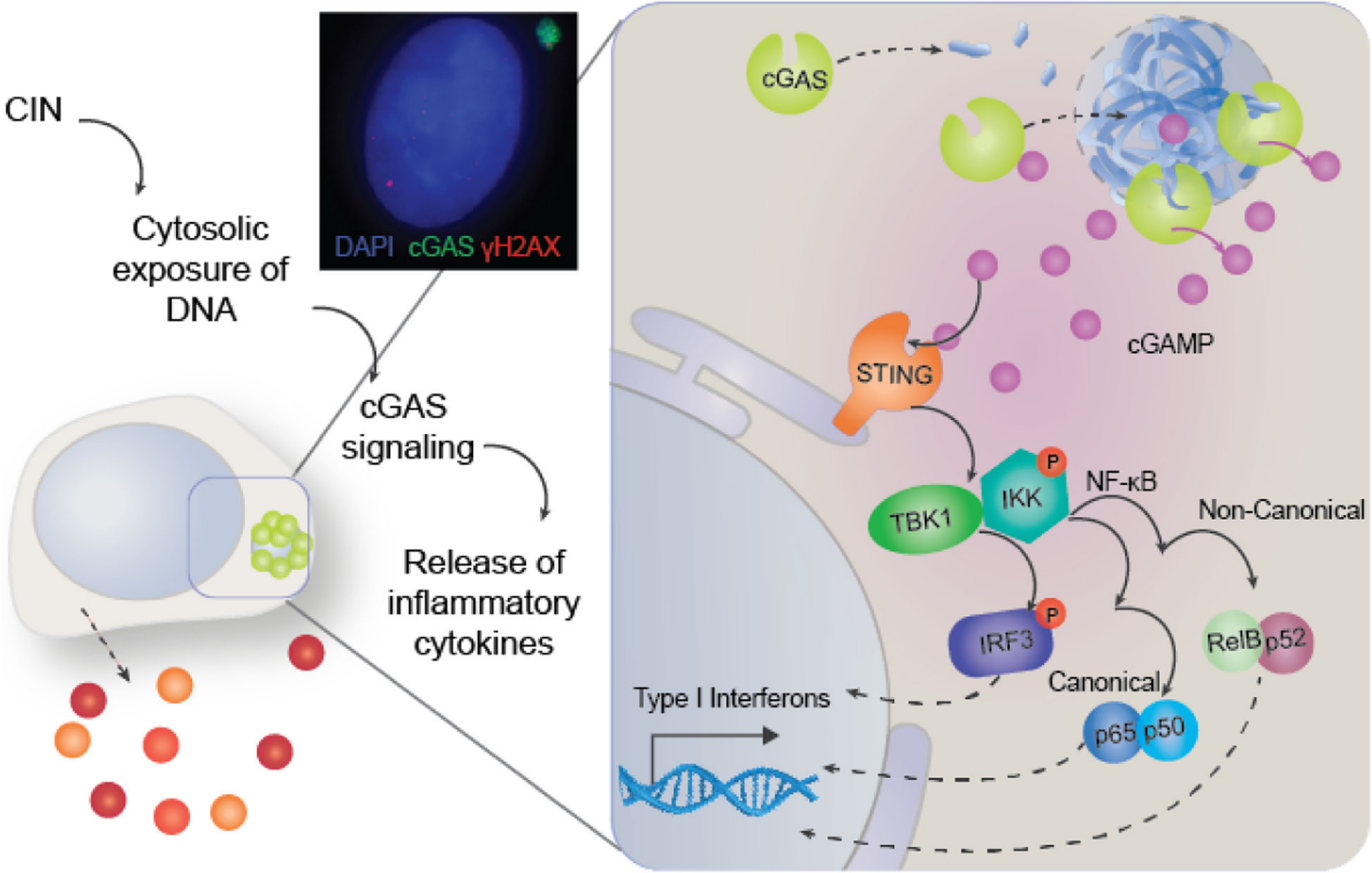
Schematic indicating the pathway from Micronucleus formation to activation of the cGAS-STING pathway, and canonical and non-canonical routes to Type I interferon response

How is this inflammatory pathway linked to CIN? Or, more concretely: how does CIN induce a cytosolic DNA response? A common characteristic of cells displaying CIN is the presence of micronuclei; small nuclear compartments often containing genetic material from lagging chromosomes. As might be expected, these micronuclear structures are not as stable as the cell nucleus itself [190]. It is therefore not surprising that research has observed regular collapse of micronuclear structures; it is estimated that up 60% of micronuclei undergo such a collapse [190]. This micronuclear collapse consequently leads to leakage of DNA into the cytosol, which would then, in turn, be recognised by cGAS, activating the cGAS-STING pathway (Fig. 3). As micronuclei and micronuclear collapse both commonly occur in CIN cancer cells, it is likely that the cGAS-STING pathway is activated in these cells. Recent research on cells with or without micronuclei observed localisation of cGAS to micronuclei, but not any other cellular structures [191]. It was determined that due to micronuclear rupture, DNA became exposed to the cytosol, leading to association of cGAS with the micronucleus and subsequent STING pathway activation [191]. This research thus links CIN with the activation of the cGAS-STING pathway and expression of type I IFNs.

The scale of the inflammation induced by the cGAS-STING pathway is exemplified by mice lacking DNAse II, which digests DNA from apoptotic cells. Such mice die in the embryonic stage due to massive inflammation from elevated cytokine expression. It was found that subsequent knockout of STING rescued the phenotype and abrogated the inflammation and lethality from DNAse II knockout [192]. This shows that the cGAS-STING pathway is also activated in response to self-DNA, and that it can mount a substantial inflammatory response [192]. One would assume that continuous leakage of DNA into the cytosol could maintain a prolonged inflammation, which could, in turn, promote tumour formation and metastasis. Indeed, it has been indicated that the cGAS-STING pathway could promote cancer formation. For example, the mutagen DMBA is able to induce skin cancer in mice. A recent study by Ahn and colleagues demonstrated that mice lacking STING did not develop nearly as many DMBA-induced tumours as wild type mice [193]. Wild type mice exposed to DMBA expressed a wide variety of inflammatory cytokines; this expression was reduced in STING knock-out mice [193]. Furthermore, DMBA was shown to cause leakage of nuclear DNA into the cytosol [193]. Together, these data indicate that DMBA-induced carcinogenesis is set in motion by DNA leakage and subsequent STING-mediated inflammation. However, even though this research provides evidence for a tumour-promoting role for the cGAS-STING pathway, further experiments by the same researchers found conflicting results. In a subsequent paper, it was uncovered that colorectal cells were found to be more susceptible to mutagen-induced colorectal cancer when these cells did not possess STING signalling [194]. In another paper, loss of STING was identified in multiple CRC samples and also associated with the development of CRC [195]. Apparently, in these cases, STING has a tumour-protective function rather than acting as a tumour promoter. This is further supported by research that found that low STING expression in gastric cancer and hepatocellular carcinoma was associated with poor prognosis [196, 197]. Of note, both these cancers are often induced by bacterial and viral infection, respectively. This may play a role in their downregulation of STING.

#### The cGAS-STING pathway in adaptive tumour immunity and metastasis

In addition, the cGAS-STING pathway is also believed to be involved in antitumour adaptive immunity, as activation of STING has been found to provoke a (CD8) T-cell response against the tumour in multiple instances [198, 199]. Interestingly, T-cell priming was found to be decreased in STING-deficient cells, further hinting towards an important role of the cGAS-STING pathway in initiating an adaptive immune response. Adding to this, injecting tumours with cGAMP was found to mount an antitumour T-cell response not only in the primary tumour, but also in already established metastases [200]. STING agonists have also been shown to reduce tumour growth when combined with other immune therapies, showing possible therapeutic potential [201, 202]. Nevertheless, research linking the cGAS-STING pathway to the occurrence of metastasis has recently been published. Experiments by Chen and colleagues indicated an important role for astrocytes in the vicinity of brain metastases. The researchers observed that brain metastases expressed cGAMP as a result of cytosolic DNA-mediated cGAS-STING activation [203]. It was argued that cGAMP was subsequently passed from brain metastases to astrocytes via gap junctions. This then led to the expression of TNF and INF-α by astrocytes, creating a prometastatic tumour environment, promoting growth and survival of brain metastases through NF-κB and IFN type I pathways [203]. Depletion of cGAS in cancer cells was found to inhibit the growth of brain metastases. This specific stromal support role seems to be specific to brain metastases, as inhibition of gap junctions reduced brain metastases, but not lung metastases [203]. Two recent studies concluded that activating STING within T-cells led to decreased proliferation and T-cell death [204, 205]. Interestingly, one of these studies found that activating STING in T-cells seemed to induce mitotic delay and a shift towards polyploidy [205]. Direct activation of the cGAS-STING pathway in T-cells could therefore also lead to reduced antitumour immunity. The prometastatic function of cGAS-STING signalling might thus only be apparent under specific conditions, explaining the contrasting results found on this matter.

Very recently, a research paper published by Bakhoum and colleagues provides evidence that links CIN with metastasis, through the activation of the cGAS-STING pathway [206]. This research comes forth from the researchers’ observation that metastases seem to have a higher rate of CIN and are more often aneuploid than their respective primary tumours. In addition, cells where CIN was suppressed (CIN-low) metastasised less often than their CIN-high counterparts [206]. They argue that a characteristic of CIN might be important for the development of metastasis. Single-cell RNAseq revealed that a large proportion of CIN-high cells expressed mesenchymal cell traits, upregulating genes that are important in metastasis and inflammation (e.g. MMP4, NF-κB pathway, and vimentin). Such cells were also shown to express more invasive behaviour in vitro [206]. It was argued that the upregulation of inflammatory genes might be due to a reaction to cytosolic self-DNA. Further experiments show that DNA that gets incorporated into micronuclei is also often found in the cytosol, indicating micronuclear collapse. Moreover, the presence of this cytosolic DNA lead to increased levels of STING, indicating cGAS-STING activation [206].

As mentioned previously, STING-mediated expression of type I IFNs has more often been associated with an antitumour effect. It is therefore curious to see cGAS-STING activation in this setting. However, in this case, the cGAS-STING pathway did not seem to initiate expression of type I IFN’s, as their expression in CIN-high cells was low and comparable to that of CIN-low cells. Instead, it was argued that STING might induce non-canonical activation of NF-κB (NC-NF-κB), as it was also found that NC-NF-κB gene targets were upregulated in CIN-high cells [206]. To more definitively show that cGAS-STING was involved in metastasis, mice injected with CIN-high cells were treated with shRNA against STING. Tumour burden, as well as metastatic dissemination was significantly reduced in these animals as assessed by bioluminescent imaging [206]. This very extensive report provides a compelling case in favour of a prometastatic role of cGAS-STING signalling and inflammation, possibly via the non-canonical activation of NF-κB. A recent paper published by Hou and colleagues may explain why STING has been shown to be involved in both pro- and anti-metastatic processes [207]. The non-canonical NF-kB pathway was seen to be activated by STING, while STING activation concurrently promoted anti-tumour immunity by inducing expression of type I IFNs (see Fig. 3). However, components of the non-canonical NF-kB pathway were seen to inhibit the expression of these IFNs [207]. The non-canonical NF-kB pathway therefore seems to be activated by the STING pathway, but could also be responsible for inhibiting its downstream components.

In addition, more details are emerging about the cGAS-STING pathway that might argue for more extensive research into the exact function of its components; specifically in the setting of human cancer. Protein structure analysis found that the structure of human cGAS differs significantly from its mouse counterpart [208]. Most interestingly, two amino acid substitutions in the protein’s DNA-binding domain have been observed to cause the human cGAS to react differently to cytosolic DNA. Human cGAS preferentially reacts to longer fragments of DNA (> 40 nt), and therefore produces less cGAMP than mouse cGAS, which was seen to also produce cGAMP upon binding with short DNA [208]. Differences between murine and human cGAS were also seen with the vascular disruptive compound DMXAA, which induced antitumour cGAS-STING activity in mice but not humans [209]. As many of the effects of the cGAS-STING pathway in cancer have been tested in mice, it is important to determine the value of such observations with this new knowledge in mind. In summary, CIN is able to induce inflammation through micronucleus rupture. The cGAS-STING pathway, responsible for this inflammation, has been implicated to have both pro- and anti-metastatic effects. Research showing that cGAS-STING is able to induce metastasis show indications that this depends on specific conditions. Further research might uncover if aneuploid or CIN cells are more adept at satisfying such conditions, leading to inflammation-driven metastasis.

### Antitumour immunity

Another factor that plays an important role in the generation of metastasis is the involvement of the host’s immune system. (*see* Table 3) Here, we aim to determine the current knowledge on the interplay between CIN and antitumour immunity.

**Table 3:** Processes and players in antitumour immunity

Apart from eradicating pathogens, an important function of the immune system is to remove cells that have the potential to become malignant. Abnormal processes in cells, such as the processes underlying CIN, will often lead to a change in antigens presented on the cells’ surface. This change can alert cells of the immune system, which will eliminate the abnormal cell; a process often termed immunosurveillance [210]. Two important types of immune cells that mediate responses against (potential) tumour cells are Natural Killer (NK) cells and cytotoxic T-cells. NK cells are cytotoxic lymphocytes that recognise and eliminate abnormal cells without the need for prior sensitisation; and are therefore part of the innate immune system [211]. NK cells are able to recognise cells with lowered or absent expression of MHC class I molecules and/or the expression of the stimulatory NKG2D receptor which is often expressed as a result of cellular stress [211]. Another important component of antitumour immunity are cytotoxic T-cells (CD8 T-cells). CD8 cells eliminate cells by recognition of specific MHC class I molecules, but have to be activated first with the help of (professional) Antigen Presenting Cells (APC) e.g. dendritic cells [212]. The interplay between cancer and the immune system comprises many more different players and factors, which is beyond the scope of this review, and has been reviewed elsewhere [212]. However, this anti-tumour immune control in itself can also sculpt the tumour population into becoming less immunogenic; a process known as immunoediting [210, 213]. Immunoediting can be seen as a process where the selective pressure of the immune system leads the tumour to eventually become less immunogenic. As a result, many cancers become effective at evading, or repressing the immune system.	

#### Antitumour immunity and metastasis

Being able to resist immune-mediated cell killing is especially vital in the case of metastasis. While the tumour microenvironment of the primary tumour can be immunosuppressive (in part due to immunoediting [214, 215]), dissemination from the primary tumour removes this protection. Cells that are able to successfully seed metastases are therefore likely to have effective immune evasion measures in place. Indeed, multiple lines of research have shown evidence that Circulating Tumour Cells (CTCs), Disseminated Tumour Cells (DTCs), and metastases seem to be more capable of resisting the influence of the immune system [216, 217]. It is known that circulating tumour cells can have a high level of CIN [218], but its implications in immune evasion have not been extensively researched. An interesting case study on ovarian cancer found that different metastatic lesions in the same patient could exhibit different levels of immune involvement. Importantly, regressing metastases showed evidence of T-cell infiltration, while progressing metastases were isolated from immune involvement [219]. These results further indicate that the immune system indeed seems to be involved in the clearing of metastases, and that progressing metastases might be successful due to their ability to evade the immune system’s involvement.

#### CIN and ITH can be immunogenic

As cells exhibiting aneuploidy and CIN can be considered abnormal, it is to be expected that aneuploid cells are recognised by lymphocytes. Indeed, inducing aneuploidy in human retinal pigment epithelial cells caused them to express factors that would be recognisable by NK cells, e.g. ULPB1/2 and CD155 [51]. In addition, mixing of these aneuploid cells with NK cells led to their effective elimination, something that was not seen with euploid cells [51]. A similar response was also seen when (near-)diploid cancer cell lines were subjected to drug-induced polyploidisation. This led to the increased expression of NK cell activating ligands and subsequent activation of NK cells [220]. Other research found that cancer cells with induced polyploidy were found to successfully grow upon implantation in lymphocyte-deficient mice, but often failed to establish in immunocompetent mice [221]. A similar phenomenon was seen when tetraploid colon organoids were only able to grow in immunodeficient mice [222]. CIN has been suggested to be a mechanism by which to generate ITH, as CIN leads to the continuous generation of new karyotypes. As a heterogeneous tumour is expected to express a wide panel of tumour antigens, it might be more vulnerable to immune clearance. It has recently been found that the immune system indeed seems to limit ITH, as fluorescently-labelled heterogeneous tumours were found to become less heterogeneous upon injection into immunocompetent mice due to the elimination of the more immunogenic subclones [223]. Thus, it seems that heterogeneous tumours might experience more pressure from the immune system.

#### CIN, ITH, and immune evasion

Knowing that the immune system recognises and limits aneuploidy, CIN and ITH, one might question how they seem to be such integral characteristics in many cancers. Even though the immune system seems to have effective ways to deal with aneuploid and heterogeneous tumours, many cancers have been seen to reciprocally develop ways to effectively evade the immune system, as discussed previously [212]. In one particular longitudinal study, it was found that metastasis persistence and recurrence were correlated with an absence of immunoediting [224]. Likely, such metastases exhibit characteristics of immune evasion. Interestingly, metastases with no evidence of immunoediting exhibited higher ploidy [224]. A comprehensive bioinformatics analysis on aneuploid tumour data performed by Davoli and colleagues found that clinical tumour samples with high aneuploidy showed reduced expression of markers of NK cells and CD8+ T-cells [225]. This indicates a lessened involvement of these immune cells in highly aneuploid tumours, and therefore, increased immune evasion in these tumours. Similarly, higher ITH has been shown to be possibly correlated to reduced immune cell infiltration in the tumour [226]. In concurrence with these observations, both CIN and ITH have been linked to reduced efficacy of immunotherapy [227–230].

A possible explanation for the lower immunogenicity of heterogeneous tumours is that tumour clearance by the immune system could be dependent on the clonal fraction of immunogenic antigen present in the tumour [231]. Recent research observed that homogeneous cell mixtures with high proportions of immunogenic antigens failed to grow when injected in mice. On the contrary, highly heterogeneous cell mixtures containing smaller proportions of immunogenic antigens were able to grow; strikingly, immune-mediated depletion of these antigens over time did not seem to occur. Earlier, it was mentioned that the immune system seems to limit heterogeneity [223]. ITH therefore could play both a promoting or repressing role in antitumour immunity, which is possibly dependent on the exact level of ITH. As CIN has been implicated to be a mechanism by which to generate genetic ITH, CIN might encourage immune evasion by inducing ITH. A recent paper has pointed towards another mechanism by which CIN could facilitate immune evasion. A possible way to avoid recognition by the immune system is by reducing or depleting the expression of neoantigens. In NSCLC, non-synonymous mutations leading to neoantigens were found to be located in areas of copy number loss more often than non-neoantigenic non-synonymous mutations, leading to the subclonal loss of previously clonal neoantigens [232]. As this effect was observed in tumour regions with low immune infiltration, it is postulated that this CIN-mediated depletion of neoantigens aids in tumour immune evasion [232]. Despite the lines of evidence showing an immunosuppressive effect of CIN and ITH, they have also been shown to be immunogenic. The immunosuppressive effects of CIN and/or ITH might therefore be dependent on several other factors.

#### Linking CIN, gene mutation, and immune involvement

Although CIN is an important factor in tumour development, one cannot overlook the importance of gene mutation, which has historically received more attention. A measure of gene mutation is mutational burden (the amount of mutations in a tumour genome); a factor distinct from CIN or SCNA burden, which is seen to influence the tumour immune infiltrate. Notably, tumours with a high mutational burden have a more tumour suppressive immune infiltrate, while aneuploid tumours seem to have a more immunosuppressive tumour environment [225, 233]. In addition, and in contrast to aneuploidy, high mutational burden has been associated with more favourable responses to immunotherapy [225, 227, 234–236]. A recent pan-cancer analysis has looked into the relationship between tumour genotype and the composition of the tumour immune infiltrate [233]. It was found that a different mutational origin of tumours (e.g. BRAF vs. RAS mutation in melanoma) was also associated with a different composition of the immune infiltrate. Furthermore, broader classifications such as overall neoantigen load and tumour heterogeneity were also associated with different immune infiltrates [233]. Interestingly, in this study high heterogeneity was associated with a more tumour suppressive immune infiltrate [233]. There are indications that somatic mutations can also work together with CIN to evade the immune system, and there are indications of a positive correlation between somatic mutation frequency and the degree of aneuploidy in patient samples, indicating that accumulating somatic mutations might still be advantageous in the setting of cancer CIN [237]. A well-known relationship between gene mutation and aneuploidy is the observation that aneuploidy is strongly associated with inactivating TP53 mutation [237, 238], most likely to allow for further chromosome mis-segregation. A recent case study discovered that a combination of copy number loss and oncogenic inactivating mutation of PTEN could have rendered a previously treatment-sensitive tumour resistant to anti PD-1 treatment [239]. These observations indicate that oncogenic somatic mutations and CIN, both separately and together, have a hand in shaping the tumour immune environment.

Presently, there is evidence both in favour of and against a role for CIN in suppressing the immune system. Even though aneuploidy, CIN, and ITH seem to be immunogenic themselves, many tumours displaying these characteristics have found ways to evade the immune system. The link between CIN and the immune system has not been researched extensively, but is beginning to be uncovered. As CIN is known to affect a wide range of cellular processes, it would be interesting to see its precise impact on stimulating or repressing antitumour immunity.

## Discussion

**Fig. 4:**
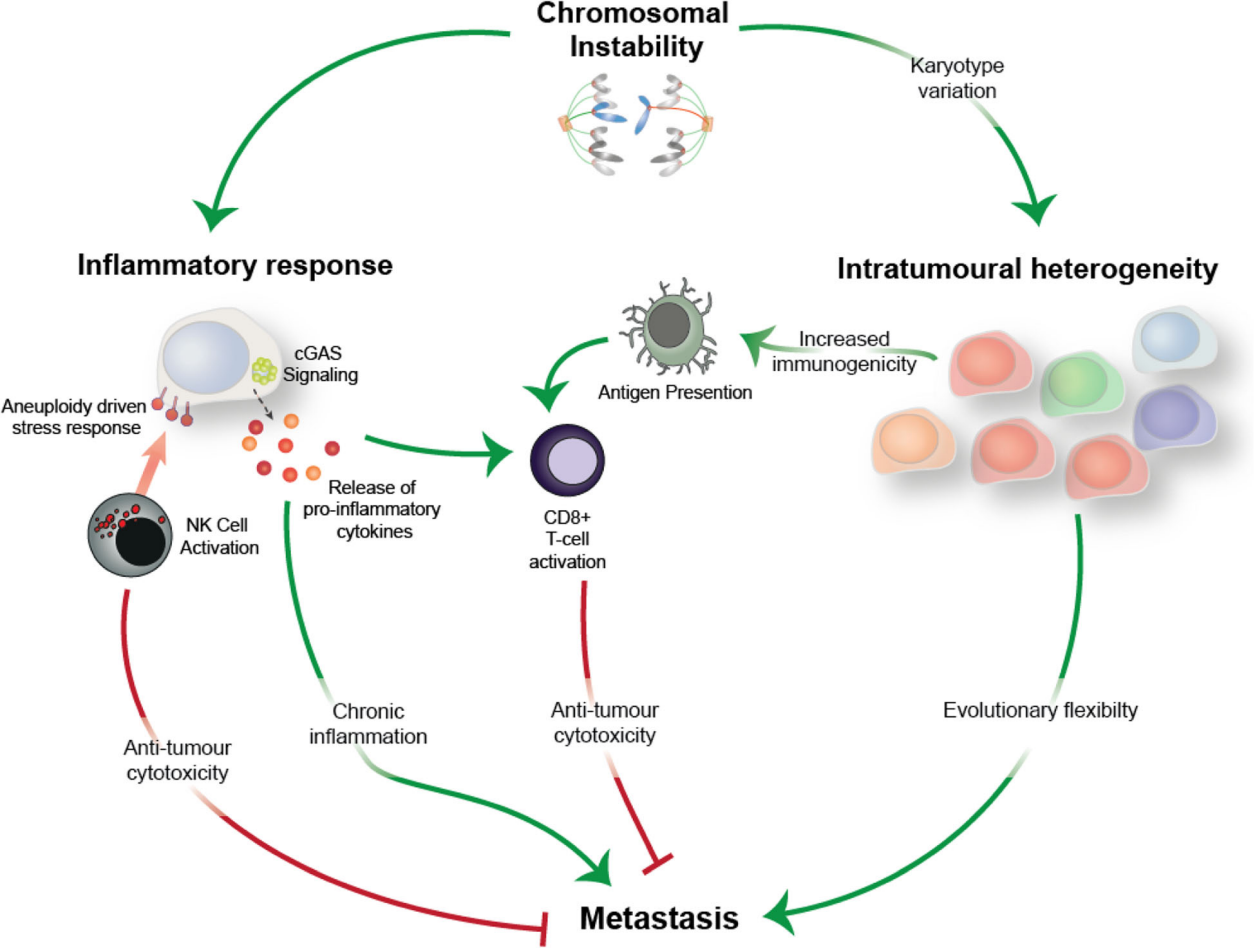
Diagram depicting the relationships between CIN, ITH, inflammation and metastasis discussed in this review

Figure 4 summarises the connections between aneuploidy, CIN, ITH, metastasis, inflammation, and tumour immunity. Though this is a long list of components, the multifactorial nature of the roles and consequences of CIN in relation to these processes means that it is important to start to look at the larger picture. While for some elements, the connection between them is clear, for other elements, the evidence for a connection between them is suggested but not yet conclusively supported by data. From the clinical research, it seems clear that there is an association between the state of aneuploidy, the process of CIN, and a worsened prognosis or higher stage. Almost all clinical research points, directly or indirectly, towards a relationship between CIN and metastasis. Recently, CIN has also been linked to inflammation by promoting the cGAS-STING pathway, but its influence in promoting metastasis has yet to be fully determined. Emerging evidence presently links CIN to both the promotion and suppression of antitumour immunity.

One of the factors that makes research on CIN and its possible influence on metastasis, inflammation and tumour immunity more difficult to investigate, is the fact that CIN is defined differently by different researchers. While some find it sufficient to take a measure of the amount of SCNAs present in a population, others use a CIN gene signature or use methods such as FISH to determine CIN. In addition, it is not clearly defined what ‘high’ and ‘low’ levels of CIN are, which could lead to discrepancies between papers. This makes it difficult to compare results of different papers and draw a general conclusion from the combined results. Future research on CIN might benefit from gaining consensus about what level of CIN can be considered ‘low’, ‘intermediate’, and ‘high’. Currently, many researchers seem to base these levels on their own data. Agreeing on such terms might aid researchers in determining the value of their observations, and may also make comparison between papers more reliable. An example suggestion for future research would be to look into tumours with higher CIN scores and not only check if they have higher rates of metastasis, but also if these tumours show higher expression of pathways associated with inflammation. Pathways of particular interest would be the cGAS-STING pathway and/or the (non-canonical) NF-κB pathway. This might then strengthen the link between CIN, inflammation, and metastasis. Recent research also suggests CIN might have an impact on antitumour immunity. As cancer immunotherapy is rising in popularity, it is important to consider the possible consequences of CIN on its efficacy.
